# Erythromycin versus neomycin in the treatment of hepatic encephalopathy in cirrhosis: a randomized double-blind study

**DOI:** 10.1186/1471-230X-13-13

**Published:** 2013-01-16

**Authors:** Fernando Gomes Romeiro, Fabio da Silva Yamashiro, Madileine Francely Américo, Luciana Aparecida Corá, Giovanni Faria Silva, JoséRicardodeArruda Miranda, Carlos Antonio Caramori

**Affiliations:** 1 Department of Internal Medicine, Faculdade de Medicina de Botucatu, UNESP – Universidade Estadual Paulista, Botucatu, Brazil; 2 Biological and Health Sciences Institute, Campus Médio Araguaia, UFMT –Universidade Federal do Mato Grosso, Barra do Garças, Brazil; 3 Health Sciences Center, UNCISAL – Universidade Estadual de Ciências da Saúde de Alagoas, Maceió, Brazil; 4 Department of Physics and Biophysics, Instituto de Biociências de Botucatu, UNESP – Universidade Estadual Paulista, Botucatu, Brazil; 5 Department of Internal Medicine – Botucatu Medical School, UNESP– Universidade Estadual Paulista, Distrito de Rubião Jr. s/n zip code 18 608 917, Botucatu, São Paulo, Brazil

**Keywords:** Hepatic encephalopathy, Liver cirrhosis, Erythromycin, Neomycin

## Abstract

**Background:**

Hepatic encephalopathy (HE) is a severe complication in patients with hepatic cirrhosis, which causes numerous hospital admissions and deaths. Antibiotics are the best options in HE treatment, but head-to-head comparisons between these drugs are scarce. Erythromycin combines the antimicrobial effect and prokinetic properties in the same drug, but it has never been used in HE treatment. Our aim was to evaluate the efficacy of erythromycin as an HE treatment.

**Methods:**

We achieved a randomized controlled trial of adult patients with HE and hepatic cirrhosis admitted in our hospital. After randomization, the subjects received either erythromycin 250 mg or neomycin 1 g orally QID until hospital discharge or prescription of another antibiotic. All subjects were blindly evaluated every day towards quantifying clinical, neuropsychometric, hepatic and renal exams. Statistical analysis was employed to compare the groups and correlate the variables with hospitalization duration.

**Results:**

30 patients were evaluated (15 treated with each drug). At hospital admission, the groups were homogeneous, but the erythromycin group subjects achieved a shorter hospitalization stay (p = 0.032) and a more expressive reduction in alanine aminotranspherase levels (p = 0.026). Hospitalization duration was positively correlated with C reactive protein levels measured previous to (p = 0.015) and after treatment (p = 0.01).

**Conclusions:**

In the sample evaluated erythromycin was associated with significant reductions in hospital stay and in alanine aminotranspherase values. Hospitalization time was positive correlated with C reactive protein levels measured before and after the treatments.

## Background

Hepatic encephalopathy (HE) is one of the major complications in patients with hepatic cirrhosis, and plays a crucial role in the prognosis. The prevalence of HE among cirrhotic patients is 30-70% according to the diagnostic criteria of overt or subclinical stages, whereas in decompensated cirrhosis the estimated incidence is 8% per year [1-3].

The occurrence of HE is debilitating and frequently results in hospitalization. In an observational study, 80% of HE episodes were received in emergency care services, and the mean hospitalization duration was between 5.7 and 7.1 days [1]. Furthermore, HE is not completely reversible in some patients, given that multiple bouts of hepatic coma constitute the only known risk factor that triggers the acquired hepatocerebral degeneration, with features suggesting toxic exposure to the brain [4].

In recent decades no major breakthrough in HE therapy has been achieved [5]. Promising results concerning benefit versus risk of antibiotics use are the most favorable [6,7]. Oral antibiotics that present low absorption can reduce the small intestinal bacterial overgrowth (SIBO) which is frequently associated with liver cirrhosis [8,9]. For this reason, antibiotics can be particularly useful in emergency settings to control ammonia production, but head-to-head comparisons in HE treatment are scarce.

Nevertheless, antibiotics commonly used in the management of HE or of intestinal bacterial illnesses play no role in motility patterns or transit time. Cirrhotic patients present many intestinal motility disorders with longer transit times when compared to normal subjects. It has been accepted that treatment with prokinetic drugs or antibiotics could improve not only the motility patterns, but also the intestinal transit, SIBO and liver function [10-12].

Erythromycin is a macrolide antibiotic that can achieve two potential targets in patients with liver cirrhosis: to reduce the intestinal bacterial overgrowth and to reorganize motility and transit time. Hence, this drug could be used to decrease the ammonia production not only in the colon but also in the small intestine, where ammonia production can occur more rapidly than in the colon in experimental studies [13]. Erythromycin estolate seems to present some advantages over the other forms [14-16] and was already evaluated in cirrhotic patients, but only at the dosage of 500 mg QID, showing no deleterious effects [17]. Minor dosages have been used as a prokinetic drug for many years, and the risk of inducing bacterial resistance is still not proven [18].

The purpose of this study was to provide a head-to-head comparison between erythromycin and neomycin in cirrhotic patients admitted to our hospital for an acute episode of HE.

## Methods

This study was approved by the local Ethics Committee and carried out according to the Declaration of Helsinki and its revisions. Informed consent was obtained.

### Patients

Thirty adult patients with confirmed diagnosis of cirrhosis and HE who had been admitted to our hospital during a two-year period between May 2008 and April 2010 participated in the study. Exclusion criteria were: severe infections, gastrointestinal bleeding, cancer, neuropsychiatric diseases, intestinal obstruction, shock, renal insufficiency (basal serum creatinine level > 2.5 mg/dl), alcohol abuse in the last six weeks, use of lactulose or any antibiotic in the preceding seven days (except as prophylactic prescription).

### Protocol

All patients received initial care focused on the precipitating factors of HE: intravenous fluids (NaCl 0.9% to achieve a urinary output above 0.5 ml/kg/h), enemas when intestinal constipation was present, and correction of electrolyte imbalances diagnosed upon hospital arrival. Subsequently, they were randomly assigned 1:1 to receive either erythromycin estolate (Eritrex® – Aché, Brazil) 250 mg QID or neomycin (Pharma Nostra Comercial LTDA, Brazil) 1000 mg QID. Both drugs were administered orally or, when necessary, through a nasoenteric tube. The drugs were maintained until hospital discharge or the prescription of another antibiotic.

**Figure 1:**
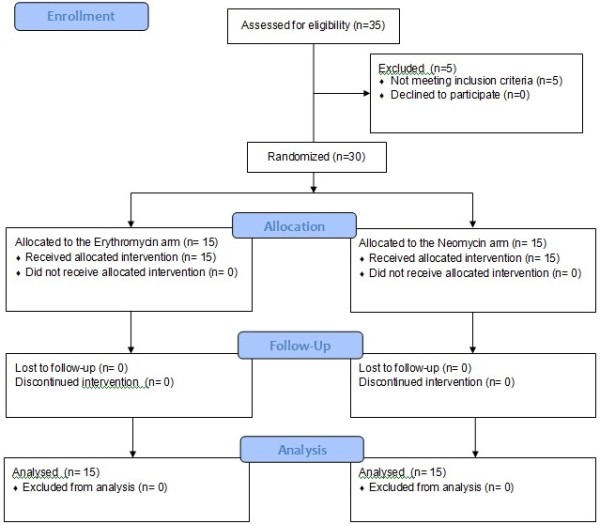
Flow diagram of patients included.

During the hospital stay each patient was managed while remaining blinded to the antibiotic treatment. When the subjects were unable to take the antibiotics a nasoenteral tube was inserted and the medications were administered through it. The tube was removed as soon as the patients were able to eat and drink safely. The diet was the same in both groups during the study. All the subjects were submitted to daily clinical exams, and to neuropsychometric and blood tests. The study flow is showed in Figure 1.

HE grading was made according to the West Haven criteria. Supplementary data were used to grade the Glasgow coma scale, flapping tremor, serum ammonia, C reactive protein (CRP), alanine aminotranspherase (ALT), aspartate aminotranspherase (AST), gamma-glutamyl transpeptidase (GGT), alkaline phosphatase (AP), urea, creatinine, and the number connection test (NCT).

The laboratory exams were performed on venous blood samples, and the ammonia quantification was made in a strict protocol: all the individuals were submitted to the same phlebotomy technique after an overnight fasting. The samples were collected, briefly maintained in a cooled recipient and immediately transported on ice to the laboratory analysis. The patients were not allowed to smoke for 8 hours before the phlebotomy. To additional comparisons between the groups, the hepatic encephalopathy index (HEI) was calculated according to previous studies, in which HEI = (HE grade x 3) + (flapping grade) + (neuropsychometric test grade) + (ammonia level grade) [19,20]. The endpoint was the hospital discharge and the results were analyzed by intention to treat.

### Statistical analysis

The results were presented as mean ± standard deviation when they showed a normal distribution and as median (first and third quartile) when not normal. Student’s t-test and Mann–Whitney test were used to compare the groups. Cirrhosis etiology, HE precipitating factors and HE classification (episodic or persistent) in the two groups were compared by Z score. The correlations between the number of in-hospital days and the other variables were measured by the Spearman rank correlation. All the data were analyzed in SigmaStat version 3.5 (Dundas software LTD, Germany) and Origin version 8.0 (OriginLab Corporation, USA). A 5% significance level was adopted.

## Results

Five of the thirty-five patients initially enrolled were excluded, mainly for infections diagnosed after the hospital admission (Figure 1). Eighteen men and twelve women composed 60% and 40% of the included individuals, respectively. According to the West Haven criteria, the sample contained ten subjects with HE grade 1, nine with grade 2, nine with grade 3 and two with grade 4. Sixteen individuals had ascites (9 in the erythromycin group and 7 in the neomycin group). The need of tube feeding was not different between the groups (4 subjects in each group).

The major etiology of cirrhosis in the sample was alcohol abuse (six subjects in each group). In seven individuals the cirrhosis etiology was not diagnosed (4 in the NEO group an 3 in the ERY group). Viral hepatitis was present in 4 subjects in the NEO group and 3 in the ERY group (six subjects presented hepatitis C and one had hepatitis B). Autoimmune hepatitis was diagnosed in 3 individuals (2 in ERY group and 1 in NEO group), and 1 subject presented alpha-1 deficiency (ERY group). The clinical and laboratory characteristics of each group are presented in Tables 1 and 2. There were no significant differences between the groups before HE treatment. The most common precipitating factors were dehydration and hyponatremia (Table 1).

**Table 1:** **Comparison of HE**-**precipitating factors between groups at hospital admission**

**Precipitating factor**	**ERY**	**NEO**	**Total**	p*
Dehydration	3	4	7 (23.0%)	1.000
Undetermined	4	3	7 (23.0%)	1.000
Hyponatremia	2	4	6 (20.0%)	0.648
Intestinal constipation	2	1	3 (10.0%)	1.000
Animal protein overload	1	1	2 (0.7%)	0.591
Urinary infection	2	0	2 (0.7%)	0.466
Benzodiazepine use	1	1	2 (0.7%)	0.591
Hypokalemia	0	1	1 (0.3%)	0.992
Total	15	15	30 (100.0%)	

ERY= erythromycin group. NEO= neomycin group. * = Z test comparison.

**Table 2:** Comparison of clinical and laboratory findings between groups at hospital admission

**Initial variables**	**Erythromycin**	**Neomycin**	**p**
Episodic HE^1^	4	7	0.449
Persistent HE^1^	11	7	0.263
Non classified HE^1^	0	1	0.992
Age (years) ^2^	55.20 ± 13.51	58.80 ± 10.47	0.421
Child-Pugh score ^3^	8.00 (8.00 : 10.50)	9.00 (8.00 : 10.75)	0.421
MELD score ^2^	16.40 ± 3.89	15.22 ± 4.40	0.440
HEG (grades) ^3^	2.00 (1.00 : 2.75)	2.00 (1.00 : 3.00)	0.421
HEI ^3^	13.00 (10.00 : 16.50)	14.00 (9.00 : 18.00)	0.588
GCS ^3^	14.00 (13.00 : 14.00)	13.00 (10.25 : 14.75)	0.394
CRP ^3^	0.80 (0.35 : 2.325)	1.40 (0.50 : 3.80)	0.475
NH_3_^3^	96.00 (80.75 : 118.50)	82.00 (45.50 : 174.75)	0.468

Child-Pugh score = Child-Pugh modified classification. HEG = hepatic encephalopathy grades. HEI = hepatic encephalopathy index. GCS = Glasgow coma scale. CRP = C reactive protein (mg/dl). NH_3 _= serum ammonia (μM/l). ^1 ^absolute values and Z score comparison; ^2 ^mean ± standard deviation and t test comparison ; ^3 ^median, 1º : 3º quartiles and Mann–Whitney test comparison.

The results of each treatment were assessed by calculating the differences between the final and the initial values of scores and exams, and are presented in Table 3. When compared to the neomycin group, the erythromycin group achieved a significant reduction in ALT levels (p = 0.026) and in hospitalization duration (p = 0.032).

**Table 3:** Comparison of the results obtained with each treatment during the hospitalizations

**Variables**	**Erythromycin**	**Neomycin**	**p**
∆ **IHD**^1^	**3**.**00** (**2**.**25 **: **4**.**00**)	**5**.**00** (**3**.**25 **: **11**.**25**)	**0**.**032**
∆ GCS ^1^	1.00 (0.25 : 2.00)	0.00 (0.00 : 3.25)	0.394
∆ MELD ^2^	−0.55 ± 1.53	−0.62 ± 2.08	0.917
∆ HEG ^1^	2 (1 : 2.5)	2 (1 : 3)	0.806
∆ HEI ^1^	−6.00 (−9.00 : -1.50)	−5.00 (−7.00 : -1.25)	0.632
∆ CRP ^1^	0.30 (0.10 : 0.70)	1.10 (0.15 : 4.30)	0.111
∆ NH_3_^2^	−31.40 ± 49.32	−30.70 ± 92.15	0.979
∆ **ALT**^2^	−**3**.**20** ± **8**.**54**	**3**.**71** ± **7**.**12**	**0**.**026**
∆ AST ^2^	−15.70 ± 48.06	8.42 ± 21.80	0.678
∆ AP ^2^	−17.70 ± 65.3	−12.30 ± 48.90	0.802
∆ GGT ^1^	−7.00 (−29.00 : 0.75)	−5.00 (−25.50 : 5.00)	0.724
∆ urea ^2^	−2.19 ± 20.64	−4.25 ± 23.72	0.802
∆ creatinine ^2^	−0.10 ± 0.27	−0.20 ± 0.30	0.289

^1 ^median, 1º : 3º quartiles ; Mann–Whitney test comparison.

^2^ mean ± standard deviation ; t test comparison.

∆ = final – initial values. IHD = in-hospital days. GCS = Glasgow coma scale. HEI = hepatic encephalopathy index. HEG = hepatic encephalopathy grade. CRP = C reactive protein (mg/dl). NH_3 _= serum ammonia (μM/l). ALT = alanine aminotranspherase, AST = aspartate aminotranspherase, AP = alcaline phosphatase, and GGT = gamma-glutamyltranspeptidase (all in U/l). Urea and creatinine are measured in mg/dl.

The correlations between the hospital stay and the variables evaluated at emergency care admission are presented in Table 4. Glasgow coma scale, HE index, HE grade and C reactive protein were significant correlated with the hospitalization duration.

**Table 4:** **Correlations between in**-**hospital days and other variables at admission**

**Variables**	**GCS**	**MELD**	**HEI**	**HEG**	**CRP**	N**H**_**3**_
IHD (R)	−**0**.**480**	−0.040	**0**.**440**	**0**.**526**	**0**.**454**	0.120
IHD (p)	**0**.**007**	0.840	**0**.**015**	**0**.**028**	**0**.**015**	0.520

IHD (R) = Spearman rank correlation coefficient with in-hospital days. IHD (p) = p value of the Spearman rank correlation with in-hospital days. GCS = Glasgow coma scale. HEI = hepatic encephalopathy index. HEG = hepatic encephalopathy grade. CRP = C reactive protein (mg/dl). NH_3 _= serum ammonia (μM/l).

**Figure 2:**
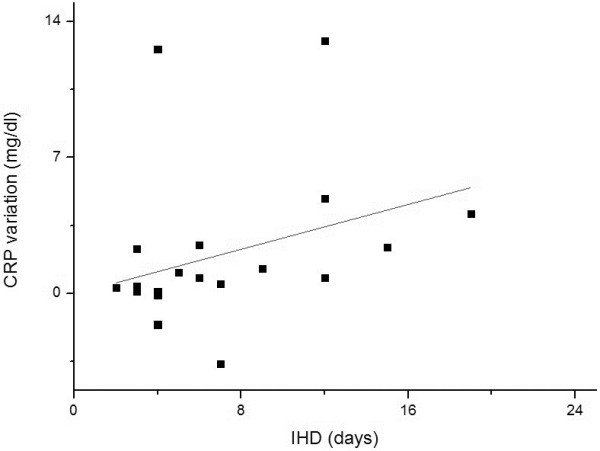
**Correlation between in-hospital days (IHD) and C reactive protein (CRP) variation.** CRP variation in each subject was calculated by subtracting the initial CRP from the final CRP value. Spearman rank correlation coefficient (R) = 0.51 and p = 0.01.

The correlations between the length of the hospital stay and the variations in C reactive protein levels obtained during the treatment are presented in Figure 2. The CRP levels were significantly correlated and varied directly with the number of in-hospital days.

### Descriptive findings

Although the sample in this study was composed of subjects with advanced cirrhosis, no adverse effects related to the antibiotics were observed. Only three subjects in the erythromycin group and six in the neomycin group had worsened their MELD scores during the treatment, with a maximum increase of 2.4 points. Two elderly individuals had acute renal injury during the study, both in the neomycin group, but in neither case was this complication attributed to the antibiotic used.

The final analysis was performed by intention to treat, but only four patients had their medications discontinued due to infection signals, which led to a shift to other antibiotics. These cases belong to the neomycin group, and they all had received the drug for at least 24 hours. Three had pneumonia and one presented a urinary infection. In color Doppler ultrasound exams, two subjects displayed signs of portal vein thrombosis and two had splenorenal shunts (one in each group in both diagnoses). Although 16 subjects had ascites there were no spontaneous bacterial peritonitis during the trial.

Only two patients were transferred to intensive care units, both in the neomycin group. There were two deaths during the study, also in this group, but the antibiotic was not implicated in any complications that occurred.

Three subjects in the erythromycin group and five in the neomycin group did not reach fifteen points on the Glasgow coma scale until hospital discharge. Excluding the two deaths in the neomycin group, two cases in each group were persisting in an alert pattern lower than the fifteen points in the Glasgow coma scale (before and after hospital admission).

Until January 2012, sixteen subjects were still alive, ten had died and four missed the follow-up. Only the younger subject in the study was submitted to liver transplantation after an episode of spontaneous bacterial peritonitis. Currently, none of them are on a transplant waiting list. Only one patient was submitted to embolization of a splenorenal shunt after several episodes of hepatic coma. The second subject suffering from this condition is still waiting for this procedure. One patient developed hepatocellular carcinoma and was submitted to transarterial chemoembolization to future inclusion in a liver transplant list. The main causes of death during the follow up were related to infections.

## Discussion

Our study has shown for the first time that erythromycin can be employed as a feasible HE treatment in cirrhotic patients. The drug has been used as an antibiotic for almost five decades and as a prokinetic for more than ten years, even in ICU patients, pregnant women and children. Its antibiotic and prokinetic effects appear to be very promising in patients with HE and cirrhosis. Our results showed significant reductions of the hospitalization duration and also of the ALT levels in patients that have been treated with erythromycin.

The use of a placebo in severe cases of HE is unjustified, and in HE treatment trials comparisons with disaccharides as controls are not adequate [7,21]. Thus, the comparison with another antibiotic enabled the correlation of our results with many other studies. We emphasize that the control group received the maximum secure doses of neomycin [2].

The most prevalent cause of cirrhosis was alcohol and the major precipitating factors were dehydration and hyponatremia, as found in other similar trials [19,22]. Most of the patients had persistent HE, and among these subjects we had some difficulty to determine the precipitating factor involved, as mentioned by other authors [23]. Besides the fact that MELD scores and Child-Pugh classifications were high, they were not correlated with the total in-hospital days, while the MELD score seems not to be associated with HE severity, as reported by other authors evaluating the renal function in similar patients [24]. This particular discrepancy between HE severity and MELD score appears to account for the low rates of liver transplantation in these patients, and could contribute to the high mortality rates caused by HE.

HE index and HE grade showed similar results, because neither ammonia levels determination nor common psychometric testing were helpful for HE grading, as already noticed by other authors [20,25,26]. As the sample was composed by many patients with severe HE, they could not collaborate properly to be submitted to other specific exams. The only way to quantify their treatment response was the clinical findings according to the West Haven criteria.

In relation to applicability, our sample was formed by subjects with more severe HE than in other studies (nine patients with HE grade 2, nine with grade 3 and two with grade 4), which is an important concern in HE trials. The option of using a new drug requires a close observation of these patients and the possibility of antibiotic switch in case of infections. Also of interest are the hospitalization duration as the study endpoint and its correlations with clinical and laboratory variables. Thus, the correlation of the number of in-hospital days with HE grade, IEH, GSC and CRP showed simple clinical aspects that could to estimate the time of HE recovery during which these patients are treated with antibiotics. Since the median in-hospital stay obtained in our trial was 5.6 days, very similar to the value found in an observational North American survey, our results would be reproducible in clinical practice [1].

The high correlation obtained between CRP levels and the total in-hospital days corroborates the impact of the systemic inflammatory status not only at the moment of hospital arrival, but also as an indicator of HE regression during the treatment with antibiotics. This finding is new and can be used to check the response to the treatment. Another study correlates the CRP levels with encephalopathy occurrence in cirrhotic patients, and many others have described the relevance of inflammation in HE neural physiopathology [24,27,28]. Nevertheless, this is the first time that CRP levels are correlated with the hospitalization duration. The role of inflammatory cytokines in affecting the blood–brain barrier and increasing the ammonia diffusion in astrocytes was also well described by other authors [29]. By associating these reports with our findings, we postulate that the CRP reduction must be a clinical target during antibiotic treatment in HE when the values obtained are high.

As also described previously, the selective decontamination of the small intestine in these patients promotes the recovery of motility and the control of bacterial products released in the blood [10,30]. Consequently, the use of prokinetics, antibiotics or probiotics can be indicated as a direct treatment against SIBO and gut dysmotility [31]. Erythromycin is a drug that combines the prokinetic and antibiotic effects to enable synergistic action in the treatment of HE. Our trial did not measure direct findings of intestinal bacterial overgrowth, but the ALT reduction can be considered an indirect indicator of the role of erythromycin in preventing the release of these bacterial products into the portal vein. According to previous studies, these patients have an increase in production and absorption of intestinal bacterial products, which can lead to a continuous flux of lipopolysaccharides and other toxic materials to the portal vein. In the liver, these substances are recognized by Kuppfer cells and thus trigger the release of tumor necrosis factor (TNF), which together with other products of these cells, leads to hepatocyte lesion [32,33]. Given that continuous hepatocyte injuries are a source of ALT elevations, we hypothesize that the decrease in the efflux of bacterial products to the liver and the control in systemic inflammation caused by them may have been the key to achieving the promising results obtained by erythromycin in this trial. To avoid inter-laboratory fluctuations, we use the differences between the final and initial ALT and CRP levels as indirect tools to document these plausible mechanisms, which could be the reason for the improvement in hepatic function obtained in other studies by the long term use of antibiotics and prokinetics in cirrhotic patients [10].

Not only SIBO must be discussed as an oligosymptomatic cause of HE, but other bacterial infections in cirrhotic patients might be important, as *Helicobacter pylori* infection. *H. pylori* infection can produce high blood ammonia concentration in these patients, and the eradication of this pathogen may be helpful for treatment and prevention of HE [34]. In this study, the subjects were not submitted to *H. pylori* tests, but the role of erythromycin could be important in infected patients. In contrast, neomycin is probably useless against bacterial infections in the stomach.

Motility disturbances are clearly associated to cirrhosis, but the subjects evaluated in this study were not submitted to motility exams. Some patients could have a better effect of erythromycin than others, and this information is important in view of future uses of erythromycin in patients with HE. According to the good results obtained with prokinetics in cirrhotic patients, erythromycin must be evaluated in other studies about this condition [10-12].

Hence, given that antibiotics are already considered the best options for HE treatment and that the new drugs are still not useful in clinical practice, our proposal is very attractive because erythromycin is well known, easy to administer and less expensive than other treatments [23]. We hope that new studies can confirm the security profile of this dosage in cirrhotic patients.

## Conclusion

In conclusion, the use of erythromycin in HE patients achieved reductions of both hospitalization length and ALT levels when compared to neomycin. Additionally, the length of hospitalization caused by HE was positively correlated with CRP levels measured before and during the antibiotic treatment. There were no adverse events that could be related to the drugs utilized, even in patients with advanced liver disease, but the latter finding needs to be confirmed in future studies.

## Competing interests

The authors declare that they have no competing interests.

## Authors’ contribution

FGR conducted the patients, performed the comparative analysis and drafted the final article. FSY conducted the patients enrolled. MFA, LAC, GFS and JRAM drafted different parts of the article. CAC participated in the comparative analysis and drafted the article. All authors read and approved the final manuscript.

## Pre-publication history

The pre-publication history for this paper can be accessed here:

http://www.biomedcentral.com/1471-230X/13/13/prepub

## References

[B1] Poordad FF Review article: the burden of hepatic encephalopathy Aliment Pharmacol Ther 2007 25 3 9 17295846 10.1111/j.1746-6342.2006.03215.x

[B2] Al Sibae MR McGuire BM Current trends in the treatment of hepatic encephalopathy Ther Clin Risk Manag 2009 5 617 626 19707277 10.2147/tcrm.s4443 PMC2724191

[B3] Moriwaki H Shiraki M Iwasa J Terakura Y Hepatic encephalopathy as a complication of liver cirrhosis: An Asian perspective J Gastroenterol Hepatol 2010 25 858 863 20546438 10.1111/j.1440-1746.2010.06242.x

[B4] Wijdicks EFM Wiesner RH Acquired (Non-Wilsonian) hepatocerebral degeneration: complex management decisions Liver Transplantation 2003 9 9 993 994 10.1053/jlts.2003.50107 12942464

[B5] Cardenas A Ginès P Management of complications of cirrhosis in patients awaiting liver transplantation J Hepatol 2005 42 S124 S133 10.1016/j.jhep.2004.12.007 15777567

[B6] Bass N Review article: the current pharmacological therapies for hepatic encephalopathy Aliment Pharmacol Ther 2006 25 Suppl 23 31 17295849 10.1111/j.1746-6342.2006.03218.x

[B7] Als-Nielsen B Gluud LL Gluud C Non-absorbable disaccharides for hepatic encephalopathy: systematic review of randomised trials BMJ 2004 328 1046 10.1136/bmj.38048.506134.EE 15054035 PMC403844

[B8] Lakshmi CP Ghoshal UC Kumar S Goel A Misra A Mohindra S Frequency and factors associated with small intestinal bacterial overgrowth in patients with cirrhosis of the liver and extra hepatic portal venous obstruction Dig Dis Sci 2010 55 1142 1148 10.1007/s10620-009-0826-0 19424796

[B9] Pande C Kumar A Sarin SK Small-intestinal bacterial overgrowth in cirrhosis is related to the severity of liver disease Aliment Pharmacol Ther 2009 29 1273 1281 10.1111/j.1365-2036.2009.03994.x 19302262

[B10] Madrid AM Hurtado C Venegas M Cumsille F Defilippi C Long-Term treatment with cisapride and antibiotics in liver cirrhosis: effect on small intestinal motility, bacterial overgrowth, and liver function Am J Gastroenterol 2001 96 1251 1255 10.1111/j.1572-0241.2001.03636.x 11316178

[B11] Park CH Joo YE Kim HS Choi SK Rew JS Kim SJ Neostigmine for the treatment of acute hepatic encephalopathy with acute intestinal pseudo-obstruction in a cirrhotic patient J Korean Med Sci 2005 20 150 152 10.3346/jkms.2005.20.1.150 15716622 PMC2808564

[B12] Gunnarsdottir SA Sadik R Shev S Simrén M Sjövall H Stotzer PO Small intestinal motility disturbances and bacterial overgrowth in patients with liver cirrhosis and portal hypertension Am J Gastroenterol 2003 98 1362 1370 10.1111/j.1572-0241.2003.07475.x 12818282

[B13] Sugarbacker SP Revhaug A Wilmore DW The Role of the Small Intestine in Ammonia Production after Gastric Blood Administration Ann Surg 1987 206 5 17 10.1097/00000658-198707000-00002 3496861 PMC1492937

[B14] Croteau D Bergeron MG Lebel M Pharmacokinetic advantages of erythromycin estolate over ethylsuccinate as determined by high-pressure liquid chromatography Aliment Pharmacol Ther 2001 15 595 603 10.1046/j.1365-2036.2001.00964.x 3259856 PMC172220

[B15] Potthast H Schug B Elze M Schwerdtle R Blume H Comparison of the bioavailabilities of erythromycin estolate and erythromycin ethylsuccinate dry suspension preparations in steady state Pharmazie 1995 50 56 60 7886126

[B16] Henry J Turner P Garland M Esmieu F Plasma and salivary concentrations of erythromycin after administration of three different formulations Postgrad Med J 1980 56 707 710 10.1136/pgmj.56.660.707 7220406 PMC2426034

[B17] Barré J Mallat A Rosenbaum J Deforger L Houin G Dhumeaux D Pharmacokinetics of erythromycin in patients with severe cirrhosis. Respective influence of decreased serum binding and impaired liver metabolic capacity Br J Clin Pharmacol 1987 23 753 757 10.1111/j.1365-2125.1987.tb03111.x 3606934 PMC1386171

[B18] Deane A Chapman MJ Fraser RJ Bryant LK Burgstad C Nguyen NQ Mechanisms underlying feed intolerance in the critically ill: Implications for treatment World J Gastroenterol 2007 13 3909 3917 17663503 10.3748/wjg.v13.i29.3909 PMC4171161

[B19] Paik YH Lee KS Han KH Song KH Kim MH Moon BS Comparison of rifaximin and lactulose for the treatment of hepatic encephalopathy: a prospective randomized study Yonsei Med J 2005 46 399 407 10.3349/ymj.2005.46.3.399 15988813 PMC2815818

[B20] Edwin N Peter JV John G Eapen CE Graham PL Relationship between clock and star drawing and the degree of hepatic encephalopathy Postgrad Med J 2011 87 605 611 10.1136/pgmj.2010.108985 21693571

[B21] Morgan MY Blei A Grüngreiff K Jalan R Kircheis G Marchesini G The treatment of hepatic encephalopathy Metab Brain Dis 2007 22 389 405 10.1007/s11011-007-9060-7 17846875

[B22] Strauss E da Costa MF The importance of bacterial infections as precipating factors of chronic hepatic encephalopathy in cirrhosis Hepatogastroenterology 1998 45 900 904 9684155

[B23] Bajaj JS Riggio O Drug therapy: Rifaximin Hepatology 2010 52 1484 1488 10.1002/hep.23866 20814894

[B24] Papadopoulus N Soultati A Goritsas C Lazaropoulou C Achimastos A Adamopouloos A Nitric oxide, ammonia, and CRP levels in cirrhotic patients with hepatic encephalopathy. Is there a connection? J Clin Gastrtoenterol 2010 44 713 719 10.1097/MCG.0b013e3181d47f71 20495469

[B25] Ferenci P Lockwood A Mullen K Hepatic encephalopathy – definition, nomenclature, diagnosis, and quantification: final report of the working party at the 11th World Congress of Gastroenterology, Vienna, 1998 Hepatology 2002 35 3 716 721 10.1053/jhep.2002.31250 11870389

[B26] Gundling F Seidl H Schmidt T Schepp W Blood ammonia level in liver cirrhosis: a conditio sine qua non to confirm hepatic encephalopathy? Eur J Gastroenterol Hepatol 2008 20 3 246 247 10.1097/MEG.0b013e3282f1d00c 18301310

[B27] Shawcross D Jalan R The pathophysiologic basis of hepatic encephalopathy: central role for ammonia and inflammation Cell Mol Life Sci 2005 62 2295 2304 10.1007/s00018-005-5089-0 16158192 PMC11139067

[B28] Seyan AS Hughes RD Shawcross DL Changing face of hepatic encephalopathy: Role of inflammation and oxidative stress World J Gastroenterol 2010 16 3347 3357 10.3748/wjg.v16.i27.3347 20632436 PMC2904880

[B29] Prakash R Mullen KD Mechanisms, diagnosis and management of hepatic encephalopathy Nat Rev Gastroenterol Hepatol 2010 7 515 525 10.1038/nrgastro.2010.116 20703237

[B30] Liu Q Duan ZP Ha DK Bengmark S Kurtovia J Riordan SM Synbiotic modulation of gut flora: effect on minimal hepatic encephalopathy in patients with cirrhosis Hepatology 2004 39 1441 1449 10.1002/hep.20194 15122774

[B31] Gupta A Dhiman RK Kumari S Rana S Agarwal R Duseja A Role of small intestinal bacterial overgrowth and delayed gastrointestinal transit time in cirrhotic patients with minimal hepatic encephalopathy J Hepatol 2010 53 849 855 10.1016/j.jhep.2010.05.017 20675008

[B32] Nolan JP The role of endotoxin in liver injury Gastroenterology 1975 69 1346 1356 1104401

[B33] Nolan JP The role of intestinal endotoxin in liver injury: a long and evolving history Hepatology 2010 52 1829 1835 10.1002/hep.23917 20890945

[B34] Chen SJ Wang LJ Zhu Q Cai JT Chen T Si JM Effect of H pylori infection and its eradication on hyperammo-nemia and hepatic encephalopathy in cirrhotic patients World J Gastroenterol 2008 14 12 1914 1918 10.3748/wjg.14.1914 18350632 PMC2700403

